# The Monday Effect: Weekly and Circadian Patterns in Acute Cardiovascular Emergencies Monday Effect in Cardiovascular Emergencies

**DOI:** 10.3390/medicina62010160

**Published:** 2026-01-13

**Authors:** Gamze Yeter Arslan, Erkan Baysal

**Affiliations:** 1 Department of Cardiology, Kepez State Hospital, 07320 Antalya, Türkiye; 2 Department of Cardiology, Gazi Yaşargil Training and Research Hospital, 21080 Diyarbakır, Türkiye; dr.erkan.baysal@hotmail.com

**Keywords:** Monday effect, acute cardiovascular events, circadian patterns

## Abstract

*Background and Objectives:* Monday syndrome refers to a psychosomatic stress response occurring at the beginning of the work week and has been implicated in triggering acute cardiovascular events. This study aimed to evaluate the association between Monday syndrome and the incidence of cardiovascular emergencies. *Materials and Methods:* Between June 2024 and June 2025, a total of 500 patients aged 18–65 years who presented with acute cardiovascular events at two centers were retrospectively analyzed. Diagnoses included STEMI, NSTEMI, unstable angina, ventricular tachycardia/fibrillation, and hypertensive emergency. The distribution of events by weekday and hour was examined. Poisson regression was used to assess the effect of Mondays on event incidence. *Results:* Of 500 patients (mean age 49.1 ± 9.7 years, 50.4% male), the most common diagnoses were STEMI (31.8%) and NSTEMI (27.4%). The incidence of cardiovascular events was highest on Mondays (19.6%) and lowest on Sundays (10.6%). The early-morning period (06:00–10:00) showed the most significant clustering of events (*p* < 0.001). Systolic blood pressure (145 ± 18 vs. 139 ± 17 mmHg, *p* = 0.008) and heart rate (87 ± 12 vs. 82 ± 13 bpm, *p* = 0.01) were significantly higher on Mondays. Monday presentation was associated with a 23% higher event rate (incidence rate ratio [IRR] 1.23, 95% CI 1.10–1.38, *p* = 0.002). *Conclusions:* Monday syndrome is associated with a significant increase in the incidence of cardiovascular emergencies, especially in the early-morning hours. Increased sympathetic tone, hormonal activation, and psychosocial stress are possible contributors.

## 1. Introduction

Cardiovascular emergencies are not randomly distributed over time but instead exhibit distinct circadian and circaseptan (weekly) patterns. A growing body of evidence has demonstrated that acute cardiovascular events including acute myocardial infarction, malignant arrhythmias, sudden cardiac death, and hypertensive crises occur more frequently during specific periods of the day and week, particularly in the early morning hours and at the beginning of the working week [1,2,3]. These temporal variations are thought to reflect complex interactions between endogenous biological rhythms and external psychosocial stressors. Similar circadian and circaseptan patterns have also been reported across a broad spectrum of cardiovascular emergencies beyond acute myocardial infarction, including arrhythmic events, hypertensive crises, and sudden cardiac death [4,5].

Among weekly patterns, the so-called “Monday effect” or “Monday syndrome” has attracted particular attention. Epidemiological studies from different populations have consistently reported a higher incidence of acute coronary syndromes and cardiovascular mortality on Mondays compared with other weekdays [6,7]. Notably, the excess risk observed on Mondays has been consistently reproduced across different countries, healthcare systems, and cardiovascular endpoints, suggesting a robust and generalizable phenomenon rather than a population-specific effect [8,9]. The transition from weekend rest to weekday work is associated with abrupt changes in sleep patterns, physical activity, and psychological stress, which may lead to increased sympathetic nervous system activation, elevated cortisol and catecholamine levels, higher blood pressure, enhanced platelet aggregability, and a prothrombotic milieu [10,11]. These pathophysiological changes are especially pronounced during the early-morning hours, when endogenous circadian rhythms already predispose individuals to cardiovascular instability. These changes are accompanied by increased cortisol release, heightened sympathetic nervous system activity, elevated blood pressure and heart rate, and enhanced platelet aggregability, collectively creating a prothrombotic and proarrhythmic milieu during the early hours of the workweek [12,13,14].

Despite extensive international data, evidence regarding weekly and circadian patterns of acute cardiovascular emergencies remains limited in Türkiye, particularly in real-world, multi-diagnostic emergency populations. Most previous studies have focused on single disease entities, such as acute myocardial infarction, and have rarely evaluated a broader spectrum of cardiovascular emergencies within the same cohort. Moreover, data examining the interaction between weekday effects and hourly presentation patterns, as well as their association with hemodynamic parameters and short-term outcomes, are scarce. In particular, data from Türkiye evaluating both circaseptan and circadian patterns across a heterogeneous emergency cardiovascular population remain scarce, highlighting an important gap in the existing literature [15,16].

Therefore, the aim of this two-center retrospective study was to investigate the association between Monday presentation and the incidence of acute cardiovascular emergencies, to characterize their circadian distribution, and to evaluate accompanying physiological and clinical features, including vital signs and short-term outcomes. By analyzing a comprehensive cohort of patients presenting with diverse acute cardiovascular conditions, we sought to provide clinically relevant insights into temporal risk patterns that may inform preventive strategies and resource planning in cardiovascular care.

## 2. Methods

### 2.1. Study Design and Setting

This two-center, retrospective observational study was conducted at Gazi Yaşargil Training and Research Hospital and Kepez State Hospital. All consecutive adult patients presenting with acute cardiovascular emergencies between June 2024 and June 2025 were screened. The study was performed in accordance with the Declaration of Helsinki and followed applicable institutional regulations for retrospective research.

### 2.2. Study Population

Patients aged 18–65 years admitted to the emergency department with an acute cardiovascular diagnosis were eligible. Acute cardiovascular emergencies were defined as ST-segment elevation myocardial infarction (STEMI), non–ST-segment elevation myocardial infarction (NSTEMI), unstable angina, ventricular tachycardia or ventricular fibrillation (VT/VF), and hypertensive emergency. Diagnoses were established based on a combination of clinical findings, electrocardiographic criteria, cardiac biomarkers, and imaging data when available, and were identified using standardized International Classification of Diseases, 10th Revision (ICD-10) codes.

For patients experiencing recurrent cardiovascular events during the same hospitalization, only the index event was included in the analysis.

### 2.3. Inclusion and Exclusion Criteria

Inclusion criteria were: (1) age between 18 and 65 years, (2) confirmed diagnosis of one of the predefined acute cardiovascular conditions, and (3) complete documentation of the date and exact time of presentation.

Exclusion criteria included: (1) trauma-related cardiovascular events, (2) iatrogenic or procedure-related complications, (3) recurrent admissions of the same patient during the study period (only the index presentation was included), (4) missing or unreliable time-stamp data, (5) incomplete clinical or laboratory records precluding adequate analysis and (6) patients with pre-hospital cardiac arrest were excluded from the analysis.

### 2.4. Data Collection and Variable Definitions

Data were retrospectively extracted from electronic hospital databases by trained investigators using a standardized data collection form. Recorded variables included demographic characteristics, cardiovascular risk factors (hypertension, diabetes mellitus, dyslipidemia, smoking status), primary diagnosis, admission vital signs (systolic blood pressure and heart rate), laboratory parameters (including cardiac biomarkers), and clinical outcomes.

The day of the week was categorized from Monday to Sunday, and time of presentation was grouped into predefined hourly intervals to evaluate circaseptan and circadian variation. Early-morning presentation was defined a priori as the interval between 06:00 and 10:00 h, based on previous circadian research.

### 2.5. Outcome Measures

The primary outcome was the incidence of acute cardiovascular emergencies according to weekday, with a particular focus on Monday presentation. Secondary outcomes included circadian distribution of events, differences in admission hemodynamic parameters, in-hospital mortality, 30-day major adverse cardiovascular events (MACE), length of hospital stay, and intensive care unit admission.

### 2.6. Statistical Analysis

Descriptive statistics were used to summarize baseline characteristics. Continuous variables were tested for normality using the Kolmogorov–Smirnov test. Normally distributed continuous variables are presented as mean ± standard deviation and were compared using Student’s *t* test or one-way analysis of variance, as appropriate. Non-normally distributed variables were summarized as medians with interquartile ranges and compared using the Mann–Whitney *U*test or Kruskal–Wallis test.

Categorical variables are expressed as frequencies and percentages and were compared using the χ^2^ test or Fisher’s exact test, when appropriate.

To evaluate the association between weekdays and the incidence of acute cardiovascular events, Poisson regression analysis was performed, and results are reported as incidence rate ratios (IRRs) with 95% confidence intervals (CIs). Multivariable models were constructed to adjust for potential confounders, including study center, age, sex, diagnostic category, and seasonal factors.

To explore circadian modification of the weekday effect, interaction terms between weekday (Monday vs. other days) and time-of-day categories were included in the regression models. Sensitivity analyses were conducted by excluding national holidays and extreme temperature days to assess the robustness of the findings.

All tests were two-sided, and a *p* value < 0.05 was considered statistically significant. Statistical analyses were performed using IBM SPSS Statistics (version 26.0; IBM Corp., Armonk, NY, USA).

### 2.7. Ethics

The study was conducted in accordance with the Declaration of Helsinki. The protocol was reviewed and approved by the Gazi Yaşargil Training and Research Hospital Clinical Research Ethics Committee (Approval No. 728, dated 7 November 2025). Due to the retrospective design and use of anonymized data, the requirement for written informed consent was waived.

## 3. Results

### 3.1. Baseline Characteristics

A total of 500 patients aged 18–65 years were included in the final analysis. The mean age of the study population was 49.1 ± 9.7 years, and 252 patients (50.4%) were male. Baseline demographic characteristics and cardiovascular risk factors are summarized in Table 1. Hypertension was the most prevalent comorbidity (46.2%), followed by dyslipidemia (32.8%) and diabetes mellitus (23.6%). Female patients more frequently had hypertension and dyslipidemia, whereas active smoking was significantly more common among male patients. Mean admission systolic blood pressure (SBP) and heart rate (HR) in the overall cohort were 142 ± 18 mmHg and 85 ± 13 beats per minute, respectively.

### 3.2. Distribution of Cardiovascular Diagnoses

The spectrum of acute cardiovascular emergencies is presented in Table 2. ST-segment elevation myocardial infarction (STEMI) was the most frequent diagnosis (31.8%), followed by non–ST-segment elevation myocardial infarction (NSTEMI) (27.4%). Together, ischemic syndromes accounted for nearly 60% of all presentations. Unstable angina represented 17.8% of cases, hypertensive emergency 14.8%, and VT/VF 8.2%. The weekday distribution of diagnoses showed a similar pattern, with ischemic events and hypertensive emergencies demonstrating a tendency toward clustering early in the week.

### 3.3. Weekly Distribution of Events

**Figure 1:**
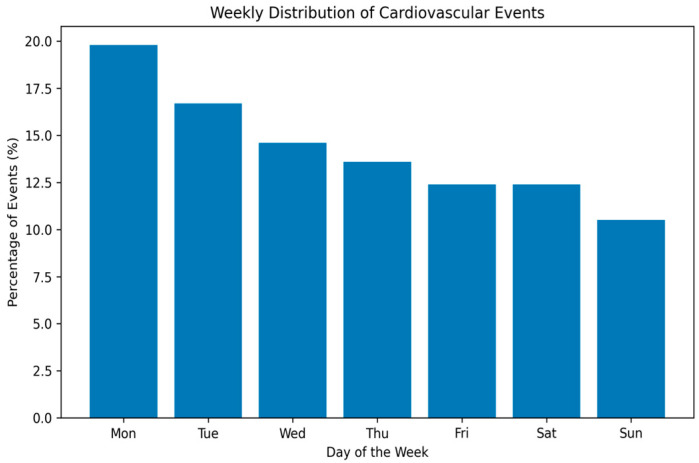
Weekly distribution of acute cardiovascular emergencies. **Legend:** Bar chart illustrating the percentage distribution of acute cardiovascular events according to the day of the week in the overall study population. A higher incidence of events is observed on Mondays, followed by a gradual decline toward the weekend.

The distribution of acute cardiovascular emergencies according to weekday is shown in Table 3 and illustrated in Figure 1. Monday accounted for the highest proportion of presentations (n = 98; 19.6%), whereas Sunday had the lowest (10.6%). In unadjusted analyses, event incidence was significantly higher on Mondays compared with other weekdays. In Poisson regression analysis adjusted for study center and relevant covariates, Monday presentation was associated with a 23% higher incidence of acute cardiovascular events (incidence rate ratio [IRR] = 1.23, 95% CI 1.10–1.38; *p* = 0.002).

Admission hemodynamic parameters differed according to weekday. Patients presenting on Mondays had higher mean SBP (145 ± 18 vs. 139 ± 17 mmHg; *p* = 0.008) and higher HR (87 ± 12 vs. 82 ± 13 beats per minute; *p* = 0.01) compared with presentations on other days.

### 3.4. Circadian Distribution and Interaction Analysis

**Figure 2:**
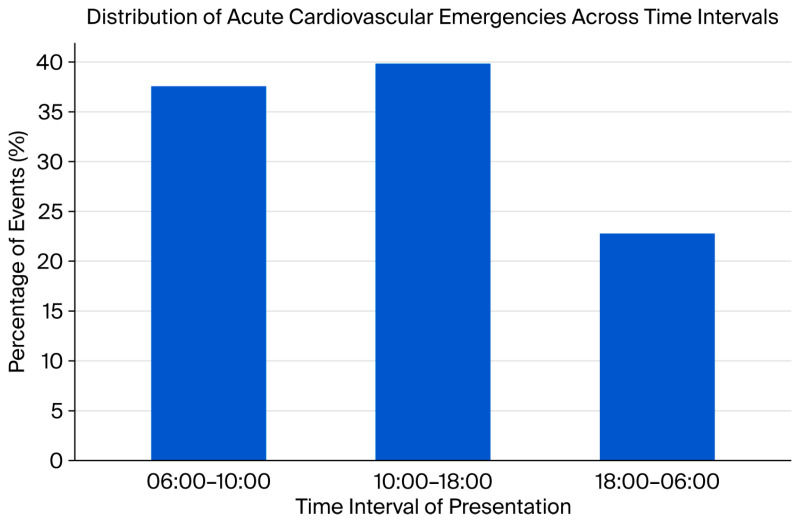
Distribution of acute cardiovascular emergencies across time intervals of presentation. **Legend:** Bar chart illustrating the percentage distribution of acute cardiovascular emergencies according to predefined time intervals of presentation. The highest incidence of events is observed during daytime hours (10:00–18:00), followed by early morning hours (06:00–10:00), with a lower incidence during nighttime hours (18:00–06:00).

Temporal distribution by hour of presentation is detailed in Table 4 and depicted in Figure 2. A pronounced circadian clustering was observed during the early-morning period (06:00–10:00), which accounted for 37.4% of all events. This time window was characterized by higher physiological stress, reflected by a higher mean HR compared with later hours of the day.

In regression models including an interaction term, a significant interaction between Monday presentation and early-morning hours was identified (IRR = 1.34; *p* < 0.001), indicating that the excess risk associated with Mondays was most prominent during the early-morning period. This pattern was consistent across sexes, with no significant effect modification by sex.

### 3.5. Short-Term Clinical Outcomes

Short-term outcomes are summarized in Table 5. In-hospital mortality was low and did not differ significantly by weekday (overall 1.2%; *p* = 0.52). However, 30-day major adverse cardiovascular events (MACE) occurred more frequently among patients presenting on Mondays compared with other days (3.0% vs. 1.8%; odds ratio 1.68, 95% CI 1.01–2.80; *p* = 0.047). Length of hospital stay and the rate of intensive care unit admission were similar across weekdays.

Sensitivity analyses excluding national holidays and extreme temperature days yielded comparable estimates for both the main Monday effect and the weekday–circadian interaction, supporting the robustness of the findings.

## 4. Discussion

In this two-center retrospective study, we demonstrated a clear circaseptan and circadian pattern in acute cardiovascular emergencies, with a pronounced excess of events on Mondays and a marked clustering during the early-morning hours. The key findings of our analysis were that (i) Monday presentations were associated with a significantly higher incidence of acute cardiovascular events compared with other weekdays, (ii) this excess risk was most prominent during the early-morning period, and (iii) Monday presentations were accompanied by less favorable hemodynamic profiles and a higher rate of short-term adverse outcomes. These findings extend previous observations by providing contemporary, real-world data from a broad emergency cardiovascular population.

Weekly variation in cardiovascular events has been consistently reported in earlier epidemiological studies, with Mondays showing the highest incidence of acute myocardial infarction and sudden cardiac death [17]. Willich et al. first described a significant Monday peak in acute myocardial infarction, attributing this phenomenon to behavioral and physiological changes associated with the transition from weekend to workweek [18]. Some investigations have extended these observations to other cardiovascular outcomes, including stroke and hypertensive emergencies, further supporting the presence of a weekday-related vulnerability pattern [19]. Subsequent studies confirmed similar patterns across different populations and cardiovascular endpoints, including hypertensive crises and arrhythmic events [20]. Our findings are in agreement with this literature and further demonstrate that the Monday effect persists across a heterogeneous spectrum of acute cardiovascular emergencies rather than being confined to a single diagnostic category. Importantly, chronobiological patterns have also been demonstrated in arrhythmic events. Remote monitoring studies in patients with cardiac implantable electronic devices have shown that atrial fibrillation and ventricular arrhythmias exhibit clear circadian and weekly variations, with an increased arrhythmic burden during the early-morning hours. These observations suggest that autonomic imbalance, sleep disruption, and psychosocial stress may modulate not only ischemic and hypertensive events but also arrhythmia occurrence, further supporting the biological plausibility of circaseptan and circadian vulnerability across diverse cardiovascular emergencies [21].

The observed interaction between weekday and circadian timing is of particular clinical relevance. Circadian vulnerability to cardiovascular events in the early morning hours has been well documented and is thought to be driven by endogenous rhythms in sympathetic nervous system activity, cortisol secretion, blood pressure, heart rate, and platelet aggregability [22]. Endogenous circadian rhythms in blood pressure regulation and autonomic nervous system balance play a central role in this vulnerability, with a well-documented morning surge in sympathetic tone and vascular reactivity [23]. The superimposition of psychosocial stress at the beginning of the workweek may amplify these physiological surges, creating a high-risk temporal window for ischemic and hypertensive events. However, individual occupational status and work schedules were not available in the present study. Therefore, the observed association between Monday presentation and increased cardiovascular events should be interpreted as hypothesis-generating rather than as evidence of a direct causal relationship with work-related stress. In our cohort, the combination of Monday presentation and early-morning hours was associated with the highest event incidence, supporting the concept of a synergistic effect between circaseptan and circadian stressors. In addition, behavioral and healthcare-seeking factors may have contributed to the observed hemodynamic differences. Delayed presentation to medical care over weekends, increased alcohol consumption, sleep deprivation, and reduced adherence to antihypertensive or other cardiovascular medications may result in higher blood pressure and heart rate at the time of hospital admission. These factors could partially explain the elevated hemodynamic parameters observed on Mondays, independent of psychosocial stress alone. Although interaction analyses were performed to explore the modification of weekday effects by time of presentation, the complete separation of circadian and circaseptan influences remains challenging in an observational study design.

Hemodynamic findings in our study provide further support for a stress-mediated mechanism. Patients presenting on Mondays had significantly higher admission systolic blood pressure and heart rate compared with other weekdays. Similar weekday-related variations in blood pressure and autonomic tone have been reported in ambulatory monitoring studies, suggesting increased sympathetic activation at the start of the workweek [24]. Elevated systolic blood pressure and heart rate are well-established triggers for plaque rupture, myocardial oxygen supply–demand mismatch, and arrhythmogenesis, providing a plausible mechanistic link between weekday-related stress and acute cardiovascular decompensation [25].

Beyond event incidence, our analysis also identified an association between Monday presentation and worse short-term outcomes, as reflected by a higher rate of 30-day major adverse cardiovascular events. Although in-hospital mortality did not differ significantly by weekday, the observed increase in early post-discharge events suggests that the physiological and clinical burden at presentation may have downstream consequences. Psychosocial stress, sleep disturbance, and autonomic imbalance have all been implicated in the pathogenesis of early adverse cardiovascular events, offering potential explanations for the higher short-term risk observed following Monday presentations [26]. Alternatively, healthcare system–related factors may also contribute to the observed increase in short-term adverse outcomes among patients presenting on Mondays. Delayed care-seeking behavior over weekends, accumulation of unresolved clinical demand, and transient overcrowding of emergency departments at the beginning of the workweek have been proposed as potential contributors to early adverse events. Therefore, the higher 30-day MACE rate observed on Mondays is likely multifactorial rather than solely attributable to biological severity at presentation. In addition to psychosocial stress, weekend-related lifestyle factors may also contribute to the observed Monday peak in cardiovascular events. Changes in dietary habits, increased alcohol consumption, sleep debt, reduced physical activity, and temporary non-adherence to chronic cardiovascular medications during weekends have all been proposed as potential triggers for early-week cardiovascular decompensation. These factors may act synergistically with circadian and circaseptan physiological stressors, further increasing cardiovascular vulnerability on Monday mornings. Prior studies examining weekday effects on outcomes have yielded mixed results, with some reporting increased early mortality or complications at the beginning of the week, while others found no significant differences [27]. Our findings add to this body of evidence by highlighting a potential prognostic relevance of temporal presentation patterns in acute cardiovascular care.

From a clinical perspective, these results may have practical implications for risk stratification and resource allocation. Awareness of heightened cardiovascular vulnerability during Monday mornings could support targeted preventive strategies, such as reinforcing medication adherence, optimizing blood pressure control, and promoting stress management and sleep hygiene at the beginning of the workweek. Medication adherence represents another important and potentially modifiable contributor to weekday variability in cardiovascular risk. Previous studies have shown that adherence to chronic cardiovascular therapies frequently declines over weekends, with missed doses commonly occurring before the start of the workweek. Simplified treatment strategies, such as the polypill approach, have been shown to significantly improve medication adherence and may help attenuate early-week surges in cardiovascular events [28,29]. In addition, emerging evidence suggests that sodium–glucose cotransporter 2 inhibitors, including empagliflozin and dapagliflozin, may reduce the risk of sudden cardiac death, highlighting the potential role of optimized pharmacotherapy in mitigating adverse temporal patterns in cardiovascular outcomes [30]. At the system level, anticipation of increased emergency cardiovascular workload during high-risk temporal windows may aid in staffing and preparedness in emergency departments and coronary care units. Large-scale studies have demonstrated a strong association between psychosocial stressors and acute myocardial infarction, underscoring the clinical relevance of stress-related cardiovascular risk at the population level [31].

### Limitations

Several limitations of this study should be acknowledged. First, the retrospective observational design inherently limits the ability to establish causal relationships between weekday or circadian patterns and the occurrence of acute cardiovascular emergencies. Associations observed in this analysis should therefore be interpreted as hypothesis-generating rather than definitive evidence of causality.

Second, the study population was limited to patients aged 18–65 years, which may restrict the generalizability of our findings, particularly to older and retired populations in whom work-related stressors may be less relevant. Cardiovascular emergencies are highly prevalent among individuals over 65 years of age, and temporal patterns in this age group may differ from those observed in a predominantly working-age cohort. Therefore, the observed Monday effect in our study should primarily be interpreted within the context of an active, working-age population.

Third, the retrospective design of the study and the reliance on electronic medical records and ICD-10 diagnostic codes may have introduced misclassification bias or incomplete data capture. Although diagnoses were supported by clinical findings, electrocardiographic criteria, and laboratory results, the possibility of coding inaccuracies or missing information cannot be entirely excluded.

Fourth, patient identification and diagnostic classification were based on electronic medical records and ICD-10 coding, which may be subject to misclassification or coding inaccuracies. Although diagnoses were supported by clinical, electrocardiographic, and laboratory data, the potential for residual diagnostic heterogeneity cannot be entirely excluded. This study was conducted at two centers and included a specific age range (18–65 years), which may limit the generalizability of the findings to older populations or to healthcare systems with different organizational structures. Additionally, unmeasured confounders such as individual work schedules, occupational stress levels, sleep deprivation, physical activity, and medication adherence could not be systematically assessed and may have influenced the observed temporal patterns.

Fifth, although multivariable and sensitivity analyses were performed to adjust for several potential confounders, residual confounding remains possible. Environmental and psychosocial stressors, including acute emotional stress, socioeconomic factors, and lifestyle behaviors preceding hospital presentation, were not directly captured in the available dataset. Therefore, residual confounding by circadian physiological variation, particularly the endogenous morning surge in blood pressure and heart rate, cannot be entirely excluded.

In addition, the lack of data on patients’ occupational status and work schedules limited our ability to directly assess the contribution of work-related stress to the observed Monday effect.

Finally, short-term outcomes were assessed, and longer-term prognostic implications of weekday and circadian presentation patterns could not be evaluated. Future prospective multicenter studies incorporating objective measures of stress, autonomic function, and long-term follow-up are warranted to further elucidate the clinical significance and underlying mechanisms of the Monday effect in acute cardiovascular emergencies. From a clinical perspective, these findings highlight the importance of temporal vulnerability in acute cardiovascular care. Recognition of increased cardiovascular risk during Monday mornings may support targeted preventive strategies, including reinforcement of medication adherence, optimization of blood pressure control, and promotion of adequate sleep and stress management at the beginning of the workweek. At the healthcare system level, awareness of predictable high-risk periods may assist in resource planning and staffing in emergency departments and coronary care units. These observations are consistent with contemporary guideline-based concepts emphasizing the role of neurohormonal activation and systemic stress in triggering acute coronary syndromes [32].

## 5. Conclusions

In this two-center retrospective study, we demonstrated a clear weekly and circadian pattern in acute cardiovascular emergencies, characterized by a pronounced excess of events on Mondays and a marked clustering during the early-morning hours. Monday presentation was associated not only with a higher incidence of cardiovascular emergencies but also with less favorable admission hemodynamic parameters and an increased rate of short-term adverse outcomes, underscoring the clinical relevance of temporal presentation patterns.

These findings suggest that the transition from weekend to workweek represents a vulnerable period for acute cardiovascular decompensation, likely mediated by the combined effects of psychosocial stress and circadian neurohormonal activation. Recognition of this high-risk temporal window may have practical implications for both individual-level prevention strategies and healthcare system planning. Reinforcing medication adherence, optimizing blood pressure control, ensuring adequate sleep, and promoting stress management, particularly at the beginning of the workweek, may help mitigate the observed excess risk.

From a systems perspective, heightened awareness of increased cardiovascular emergency burden on Monday mornings could inform staffing, resource allocation, and preparedness in emergency departments and coronary care units. Future prospective multicenter studies incorporating objective measures of stress and autonomic function, along with long-term follow-up, are warranted to further elucidate the clinical significance and underlying mechanisms of the Monday effect in acute cardiovascular emergencies.

## Figures and Tables

**Table 1:** Baseline characteristics and comorbidities.

Variable	Total (n = 500)	Female (n = 248)	Male (n = 252)
Age (years, mean ± SD)	49.1 ± 9.7	48.7 ± 9.9	49.5 ± 9.5
Hypertension	46.2%	53.2%	39.3%
Diabetes mellitus	23.6%	27.4%	19.8%
Dyslipidemia	32.8%	36.7%	29.0%
Smoking	41.4%	27.8%	54.7%
Family history of CAD	19.2%	21.0%	17.5%

**Legend:** Baseline demographic characteristics and cardiovascular risk factors of the study population. Continuous variables are presented as mean ± standard deviation, and categorical variables as number (percentage).

**Table 2:** Diagnostic distribution.

Diagnosis	n	%
STEMI	159	31.8
NSTEMI	137	27.4
Unstable angina	89	17.8
VT/VF	41	8.2
Hypertensive emergency	74	14.8

**Legend:** Distribution of acute cardiovascular diagnoses in the overall study population. Values are presented as number (percentage).

**Table 3:** Weekly distribution of cardiovascular events.

Day	n (%)	Mean SBP (mmHg)
Monday	98 (19.6)	145 ± 18
Tuesday	84 (16.8)	142 ± 17
Wednesday	73 (14.6)	141 ± 16
Thursday	68 (13.6)	139 ± 18
Friday	62 (12.4)	140 ± 17
Saturday	62 (12.4)	139 ± 18
Sunday	53 (10.6)	138 ± 17

Distribution of acute cardiovascular emergencies according to the day of the week. Values are presented as number (percentage). SBP indicates systolic blood pressure. Comparisons were performed using the chi-square test or Student’s *t*-test, as appropriate.

**Table 4:** Hourly distribution of events.

Hour Interval	n (%)	Mean HR (bpm)
06:00–10:00	187 (37.4)	88 ± 12
10:00–18:00	199 (39.8)	83 ± 13
18:00–06:00	114 (22.8)	82 ± 11

Legend: Hourly distribution of acute cardiovascular emergencies according to time of presentation. Values are presented as number (percentage). HR indicates heart rate. Comparisons were performed using the chi-square test or Student’s *t*-test, as appropriate.

**Table 5:** Short-term clinical outcomes according to weekday of presentation.

Outcome	Monday (n = 98)	Other Days (n = 402)	*p* Value
In-hospital mortality, n (%)	1 (1.0)	5 (1.2)	0.52
30-day MACE, n (%)	3 (3.1)	7 (1.7)	0.047
ICU admission, n (%)	12 (12.2)	41 (10.2)	0.41
Length of hospital stay (days)	5.6 ± 2.1	5.3 ± 2.0	0.28

**Legend:** Short-term clinical outcomes of patients presenting on Mondays compared with other weekdays. Values are presented as number (percentage) or mean ± standard deviation, as appropriate. MACE indicates major adverse cardiovascular events; ICU, intensive care unit.

## Data Availability

The data supporting the findings of this study are available upon reasonable request.
